# KRAS-Driven Metabolic Rewiring Reveals Novel Actionable Targets in Cancer

**DOI:** 10.3389/fonc.2019.00848

**Published:** 2019-08-30

**Authors:** Emanuela Pupo, Daniele Avanzato, Emanuele Middonti, Federico Bussolino, Letizia Lanzetti

**Affiliations:** ^1^Department of Oncology, University of Torino Medical School, Turin, Italy; ^2^Candiolo Cancer Institute, FPO-IRCCS, Turin, Italy

**Keywords:** KRAS, PDAC, metabolic rewiring, metabolic adaptability in cancer, NSCLC, gluocose metabolism in cancer, glycolysis

## Abstract

Tumors driven by mutant KRAS are among the most aggressive and refractory to treatment. Unfortunately, despite the efforts, targeting alterations of this GTPase, either directly or by acting on the downstream signaling cascades, has been, so far, largely unsuccessful. However, recently, novel therapeutic opportunities are emerging based on the effect that this oncogenic lesion exerts in rewiring the cancer cell metabolism. Cancer cells that become dependent on KRAS-driven metabolic adaptations are sensitive to the inhibition of these metabolic routes, revealing novel therapeutic windows of intervention. In general, mutant KRAS fosters tumor growth by shifting cancer cell metabolism toward anabolic pathways. Depending on the tumor, KRAS-driven metabolic rewiring occurs by up-regulating rate-limiting enzymes involved in amino acid, fatty acid, or nucleotide biosynthesis, and by stimulating scavenging pathways such as macropinocytosis and autophagy, which, in turn, provide building blocks to the anabolic routes, also maintaining the energy levels and the cell redox potential (1). This review will discuss the most recent findings on mutant KRAS metabolic reliance in tumor models of pancreatic and non-small-cell lung cancer, also highlighting the role that these metabolic adaptations play in resistance to target therapy. The effects of constitutive KRAS activation in glycolysis elevation, amino acids metabolism reprogramming, fatty acid turnover, and nucleotide biosynthesis will be discussed also in the context of different genetic landscapes.

## Introduction

*KRAS* mutations can promote all the key aspects of cancer cell metabolism. It elevates glucose, glutamine and fatty acids uptake and consumption to sustain biosynthetic pathways and the cell redox potential. All these functions are regulated by a number of events, here summarized in three major points, that cooperate with mutant Kras in metabolic reprogramming and specify metabolic adaptation in different tumor types.

(i) Similarly to other oncogenic lesions (2), the effect of *KRAS* mutations in metabolic adaptation can differ in distinct tumor types depending on the tissue of origin. This has been revealed by comparing the metabolic adaptations of non-small cell lung carcinoma (NSCLC) and pancreatic ductal adenocarcinoma (PDAC) driven by *Kras* mutations and *Trp53* deletion in mice. These two cancer types, despite sharing the same genetic alteration, use branched-chain amino acids differently. While NSCLCs incorporate free branched-chain amino acids into tissue protein and use them as nitrogen source, uptake of these amino acids and expression of key enzymes responsible for their catabolism are decreased in PDACs (3).

(ii) Cancer cells carrying mutant Kras crosstalk with the microenvironment, exchanging cytokines, growth factors, and metabolites to improve metabolic adaptation and overcome low nutrients availability (4–6).

(iii) Finally, a number of concomitant genetic alterations have been shown to cooperate with *KRAS* mutations in sustaining specific metabolic adaptations (7–10).

In this framework, the purpose of this review is to discuss the most recent findings on the interplay between Kras and metabolism focusing on metabolic dependencies of mutant Kras-driven lung and pancreatic cancers that could be attractive as therapeutic targets.

## Mutant KRAS and Glucose Metabolism

**Figure 1:**
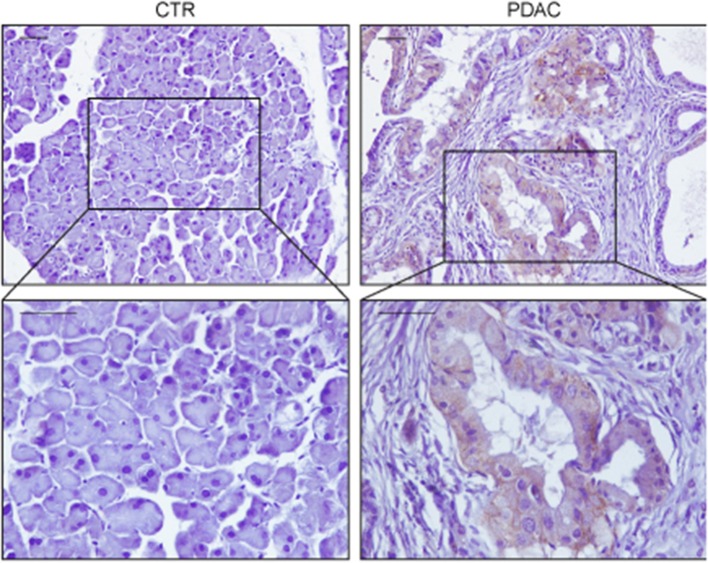
Representative immunohistochemistry stainings of GLUT1 in sections of pancreas from a wild type mouse (CTR) or from a mouse expressing Kras^G12V^ in the acinar/centroacinar lineages (Elas-tTA/tetOFF-Cre;K-Ras^+/LSL G12V Geo^) (18). GLUT1 is up-regulated specifically in most tumor cells, with mixed membranous/intracellular localization. In each case pancreas was formalin fixed, paraffin embedded and slices were processed as described in Pupo et al. (19). Briefly, paraffin removal was performed with two 10 min steps in Xylene, rehydrated in decreasing concentration of ethanol, and antigen retrieval was performed using 2100 Antigen Retriever/R-Universal buffer (Aptum Biologics). Slices were permeabilized with 0.2% TritonX, saturated in 5% goat serum/BSA and endogenous peroxidase was inhibited by H_2_O_2_ incubation. Staining was performed with anti-GLUT1 antibody (AbCam, 1:200) and secondary antibody anti-Rabbit-HRP (Dako). Immunoreactivity was developed using DAB chromogen (Dako). Scale bars are 50 μm.

The involvement of the Ras oncogene in metabolic reprogramming has been initially revealed by its ability to promote glycolysis (11). In pancreatic cancer, *KRAS* mutations are an early event being detectable in the initial lesions known as pancreatic intraepithelial neoplasias (PanIN), which can progress in infiltrating ductal carcinomas through the acquisition of additional genetic alterations (12). In mouse models, PanIN lesions rapidly evolve in aggressive PDACs when *Kras* mutations are combined with *Trp53* loss (13). Elevation of glycolysis is a distinguishing feature of Kras-driven tumorigenesis. Indeed, in the Kras mutant NSCLC model, inhibition of increased lactate production, which results from high rates of glycolysis, severely impacts on disease progression (14). Moreover, increased expression of the facilitative glucose transporter GLUT1, which fosters glycolysis by increasing glucose uptake (15), can be invariably detected in Kras mutant pancreatic lesions (16, 17) (Figure 1). The major outcome of increased glycolysis is the generation of intermediates that can be used as building blocks by other metabolic routes to synthetize nucleotides, amino acids, and fatty acids which are required by the rapidly dividing cells to generate the tumor mass (20). Indeed, elevation of glycolysis by Kras channels glucose intermediates in the pentose phosphate pathway (PPP) and in the hexosamine biosynthesis pathway (21). Using a Kras^G12D^ inducible PDAC murine model (also carrying deletion of p53), abrogation of Kras^G12D^ expression causes tumor regression that is accompanied by severe reduction of the expression of GLUT1 and rate-limiting glycolytic enzymes, and of the amount of glycolytic intermediates as revealed by both metabolomics and transcriptomic studies (21). These metabolites fuel the non-oxidative arm of PPP whose primary function is to produce the nucleotide precursor ribose-5-phosphate. Mechanistically, activation of MAPK by Kras up-regulates Myc-directed transcription. In turn, this increases the expression of the glycolytic enzymes that promote glucose uptake and consumption, and of the PPP enzyme RPIA. RPIA catalyzes the conversion of ribose-5-phosphate in ribulose-5-phosphate, thus fueling nucleotides biosynthesis (21, 22). In agreement, inhibition of PPP suppresses xenograft tumor growth indicating that mutant Kras, by increasing glucose uptake and consumption, sustains biosynthetic pathways leading to nucleotide production finally maintaining tumor growth (21). Interestingly, nucleosides supplementation can rescue cell death caused by Kras knockdown in mutant Kras-addicted PDAC cell lines without promoting cell proliferation suggesting that the metabolic function of Kras can be uncoupled from its functions in proliferation (22).

The genetic landscape of the tumor cooperates with *KRAS* mutations in the elevation of glycolysis to promote cancer growth and dissemination. In pancreatic cancer, overexpression of paraoxonase 2 (PON2), a target of p53 transcriptional repression, has been found to join forces with mutant Kras to elevate glycolysis. PON2 increases glucose uptake by binding to GLUT1 thus preventing interaction of the latter with the inhibitory protein STOM (7). PON2 overexpression controls the cell starvation response and increases glucose uptake to protect pancreatic cancer cells from detachment-induced cell death, which, in part, occurs through suppression of the AMPK/FOXO3A/PUMA signaling pathway (7). AMPK is a highly conserved kinase that works as a sensor of low cellular energy and that can either repress or promote tumor growth depending on tumor type and context (23). Here, pharmacological activation of the AMPK pathway inhibits growth of tumors generated by subcutaneous injection of PDAC cancer cells revealing a potential metabolic druggable vulnerability (7).

## Scavenging Pathways and Amino Acid Metabolism in KRAS Mutant Cancer Cells

*KRAS* mutations are known to stimulate processes such as macropinocytosis and autophagy that can scavenge nutrients from, respectively, external and internal compartments to sustain cancer cell survival under condition of nutrient deprivation [reviewed in Kimmelman (1)]. Both these two scavenging pathways generate vesicles, macropinosomes, and autophagosomes, which ultimately fuse with lysosomes to release their cargoes for degradation. In the lysosomes, breakdown of nutrients provides the cell with pools of free amino acids, lipids, nucleotides and glucose that can be used by the anabolic pathways for synthetizing novel macromolecules (1, 24). Interestingly, both in Kras mutant lung and pancreatic cancers, the lysosomal compartment undergoes expansion thanks to the increased activity of the transcription factors Tfeb/Tfe3 (25, 26), which are responsible for lysosomal biogenesis (27, 28). In Kras-driven NSCLC, glucose starvation activates AMPK that promotes dephosphorylation and nuclear translocation of Tfeb and Tfe3 (25). Accordingly, Tfe3 activity is required for growth of mouse lung tumors and increased expression of lysosomal genes correlates with accelerated disease recurrence in human lung adenocarcinoma patients (25). Similarly, upregulation and increased nuclear residence of Tfe3 sustain pancreatic tumor growth (26). Of note, overexpression of Mitf, which belongs to this family of transcription factors, promotes progression of Kras mutant PanIN lesions in PDAC indicating that increased lysosomal activity plays a driver function in mutant Kras tumors (26).

**Figure 2:**
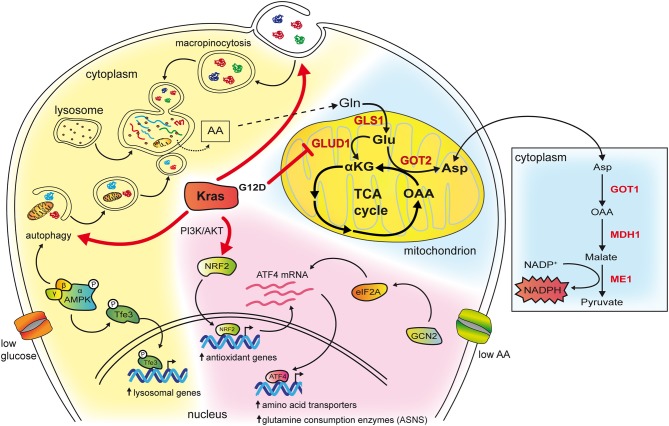
The cartoon schematizes some of the effects of mutant Kras in reprogramming amino acid metabolism. The cytoplasm of the cell has been colored in blue in the background to highlight the role of mutant Kras in PDAC, in pink to represent pathways revealed in NSCLC, in yellow when the mechanisms are common to both tumor types. Kras potentiates both macropinocytosis and autophagy whose vesicles end up in lysosomes, a compartment that is frequently found enlarged in Kras mutant cancer cells. In, NSCLC, in condition of low glucose, this occurs by activating AMPK that phosphorylates Tfe3 resulting in its nuclear translocation and transcription of lysosomal genes. Moreover, AMPK activation increases autophagy initiation and maturation. Breakdown of macromolecules in lysosomes produce free amino acids available to biosynthetic and energy pathways. Among them glutamine can enter the TCA cycle in mitochondria (depicted on the right). In PDAC glutamine is consumed through an alternative pathway (highlighted in red and in the box on the right). In NSCLC, mutant Kras activates the PI3K/AKT pathway that, in condition of low glutamine, favors mRNA expression of the ATF4 transcription factor via the NRF2 factor. In addition, NRF2 is also a key regulator of genes involved in the antioxidant response. Under condition of asparagine deprivation, the GCN2-eIF2 pathway prompts transduction of the ATF4 mRNA into protein, which, in turn, activates the transcription of amino acids transporters and glutamine consuming enzymes. Among them, asparagine synthetase ASNS catalyzes the synthesis of asparagine from glutamine. Asparagine levels and ASNS control proliferation, mTORC1 activation and suppress apoptosis.

Macropinocytosis is a non-selective actin-dependent endocytic process that uptakes nutrients from the extracellular environment in large intracytoplasmatic vesicles (29). In tumors, macropinocytosis works as a feeding mechanism to overcome high nutrients demand and support metabolic flexibility and adaptation. *KRAS* mutations have been shown to stimulate macropinocytosis allowing for large uptake of albumin, the most abundant serum protein, which is degraded in lysosomes to increase the intracellular pool of amino acids (30, 31). Breakdown of albumin provides amino acids that feed the central carbon metabolism (30) and, among them, glutamine, is avidly used by Kras transformed cells for anaplerosis and nucleotide production (30, 32) (Figure 2). Indeed, in Kras mutant pancreatic cancer cells, glutamine is the major carbon source and is consumed via a non-canonical pathway. In the majority of non-transformed cells, in mitochondria, glutamine-derived glutamate is converted, by the enzyme glutamate dehydrogenase (GLUD1), in α-ketoglutarate to fuel the tricarboxylic acid (TCA) cycle. Instead, in PDAC cells, glutamate is used by the mitochondrial aspartate transaminase GOT2 to produce aspartate and α-ketoglutarate. Aspartate is transported in the cytoplasm where it is converted to oxaloacetate, by the aspartate transaminase GOT1, then into malate and pyruvate thus elevating the NADPH/NADP^+^ ratio, which, in turn, sustains the cell redox potential (33) (Figure 2). In agreement, genetic deletion of any enzyme in the pathway elevates production of reactive oxygen species, diminishes the amount of reduced glutathione, and results in suppression of PDAC growth both *in vitro* and *in vivo* (33). Kras drives the alternative glutamine consumption pathway by up regulating transcription of GOT1 and reducing expression of GLUD1. While this pathway is essential for PDAC growth, it seems to be dispensable in non-transformed cells. This offers a therapeutic option to this type of tumors also considering that its inhibition might synergize with therapies that increase intracellular reactive oxygen species such as chemotherapy and radiation (33). Along this line, Kras mutant cells that have become resistant to cisplatin, a compound that works by increasing the reactive oxygen species in the cytoplasm, display elevation of glutamine consumption and anti-oxidant capacity (34). Knock down of GOT1 in the resistant cells reduces their proliferation suggesting that Kras-mediated metabolic reprogramming of glutamine consumption contributes to the acquired resistance to platinum-based drugs (34).

The role of Kras in detoxification is also reported in advanced lung cancer, where high frequency of *Kras*^*G12D*^ copy gain is observed. This enrichment in mutant alleles promote channeling of glucose-derived metabolites in the TCA cycle and glutathione biosynthesis enhancing the management of reactive oxygen species and increasing the metastatic potential (35). It is of note that upregulation of glutathione is specifically associated with increased mutant gene copy number highlighting a “dose” effect and suggesting therapeutic vulnerability (35).

Macroautophagy (here referred as autophagy) promotes survival under metabolic stress conditions by directing intracellular components to lysosomes via the formation of vesicles known as autophagosomes (24). Even if autophagy does not increment the biomass, as it re-utilizes pre-existing molecules to generate new ones, it supports cell survival under stress condition allowing tumor persistence (36). Autophagy is known to sustain several aspects of Ras transformation, from maintenance of the cell glycolytic capacity (37), of the mitochondrial oxidative metabolism (38), of energy charge and nucleotide pool (39), to the secretion of pro-migratory cytokines (40). Autophagy has complex functions in cancer, being both pro-tumorigenic and tumor suppressive (24), but increasing evidence in mouse models of pancreatic cancer indicates that, especially at later stages of tumorigenesis, autophagy sustains tumor growth [reviewed in Amaravadi and Debnath (41)]. Indeed, pancreatic deletion of the autophagy gene *Atg5* in a model of pancreatic cancer driven by oncogenic *Kras* and the stochastic loss of heterozygosity of *Trp53* (*Kras*^*G12D*^; *Trp53*^*lox*/+^), a condition that reproduces the stepwise human development of pancreatic cancer, increases the number of PanIN lesions, but impairs the progression of PanIN to PDAC, prolonging mice survival (42). Moreover, inhibition of autophagy by treatment with hydroxycloroquine causes tumor reduction in *KRAS* mutant *TP53* mutant patients-derived pancreatic cancer xenografts (42). In addition, the effects of intermittent autophagy inhibition, which would mimic patients treatment, have been recently tested using an inducible transgenic PDAC mouse model generated by crossing mice carrying the inducible dominant-negative mutant of the autophagic gene *Atg4B* with the *Kras*^*G12D*^; *Trp53*^*lox*/+^, mice. In these animals, metronomic impairment of autophagy has been found to delay tumor growth via both cell autonomous, by decreasing proliferation and sensitizing apoptosis in nutrient-restricted areas of the tumor, and non-autonomous, macrophage-mediated, mechanisms (5).

Notably, two recent studies have shown that autophagy inhibition synergizes with pharmacological targeting of the KRAS downstream effectors MEK1/2 or ERK, preventing growth of KRAS-driven pancreatic adenocarcinomas (43, 44). The efficacy of combining these two treatments appears to rely on the fact that inhibition of the MAPK pathway, one of the major pathways downstream KRAS, potentiates autophagy, suggesting that this treatment causes addiction to autophagy. Concomitant treatment with MAPK and autophagy inhibitors might therefore represent a novel strategy to target KRAS-driven cancers (43, 44).

The ability of mutant Kras to model the microenvironment is a long standing observation in PDACs where abrogation of Kras^G12D^ expression, not only affects tumor growth, but also reduces the desmoplastic stroma, which is typical of this type of cancer (18). In PDACs, mutant Kras instructs the microenvironment to sustain tumor growth both by engaging stromal cells that instigate reciprocal signaling (4), and by exploiting stroma-derived alternative fuels (6). This latter function relies on the stroma-associated pancreatic stellate cells that, following stimulation by the cancer cells, activate autophagy and secrete their breakdown products mainly consisting of non-essential amino acids. Among them, alanine, the second most abundant amino acid in proteins, is up-taken by the cancer cells and used as carbon source to run the TCA cycle, and to synthetize other non-essential amino acids and lipids (6).

The role of Kras in mediating the nutrient stress response to reduced amino acid availability has been recently elucidated in NSCLC. Gene expression profiles of lung cancer cell lines with different genetic background have been analyzed in presence of high or low glutamine concentrations with or without concomitant Kras knockdown, to identify a set of genes that are differentially regulated by Kras signaling in response to glutamine availability (45). In low glutamine, Kras regulates over 100 genes. Among them, 39 are controlled by the transcription factor ATF4. Kras increases the expression of ATF4 mRNA through PI3K-AKT-mediated upregulation of the NRF2 transcription factor, which drives the expression of a number of genes mainly involved in the antioxidant response [reviewed in Sullivan et al. (46)]. During nutrient deprivation, activation of the GCN2-p-eIF2 pathway stimulates translation of the ATF4 mRNA, resulting in increased ATF4 protein levels and transcription of target genes responsible for amino acids uptake and metabolism thus regulating cell proliferation and mTORC1 activation (45). Among the ATF4 targets, the enzyme asparagine synthetase (ASNS), which transfers the γ amino group of glutamine to aspartate, yielding asparagine and glutamate, uncovers a key role because it contributes to apoptotic suppression, protein biosynthesis and mTORC1 activation. Consistently, inhibition of AKT impairs Kras-dependent activation of ASNS therefore sensitizing NSCLC tumors to depletion of extracellular asparagine (45). Overall these findings identify KRAS as a master regulator of the transcriptional response to nutrient deprivation that controls amino acids uptake and consumption (schematized in Figure 2). ATF4 has been shown to exert both pro- and anti-oncogenic effects depending on the genetic context and nutrient availability (45). In condition of low glutamine, ATF4 has a protective role toward apoptosis in Kras mutant NSCLC cell lines that carry loss of *KEAP1* (45), a deletion that, in humans, affects approximately 20% of Kras-mutant lung adenocarcinomas (8). Keap1 is a ubiquitin ligase that causes degradation of NRF2 [reviewed in Sullivan et al. (46)]. Its loss cooperates with *KRAS* mutations in lung adenocarcinoma progression by opposing to the oxidative stress barriers during tumorigenesis (8). Of note, Kras mutant Keap1 deficient cancers are dependent on the glutamine anaplerotic pathway as their growth rate in mice is reduced by pharmacological inhibition of the enzyme glutaminase. This suggests that increased NRF2 activation in Kras mutant lung cancer might be exploited as a stratification tool to identify patients that benefit from glutaminase inhibition (8).

In NSCLC, *KRAS* mutations are often accompanied by loss of the tumor suppressor *STK11*, which encodes the LKB1 kinase, leading to the formation of aggressive tumors characterized by perturbed nitrogen handling (9). LKB1, through AMPK, suppresses transcription of CPS1, (carbamoyl phosphate synthetase-1), a mitochondrial enzyme that catalyzes the rate-limiting step of the urea cycle. In non-pathological settings, expression of CPS1 is restricted to the liver where robust urea production from ammonia takes place (47). In NSCLC cells bearing both mutant Kras and LKB1 loss, expression of CPS1 produces carbamoyl phosphate in the mitochondria from ammonia and bicarbonate, initiating pyrimidine synthesis (9). Depletion of CPS1 in these cells results in pyrimidine depletion, replication fork stalling and DNA damage finally reducing their ability to grow tumors. Interestingly, wild type Kras cells carrying LKB1 loss express CPS1, but do not depend on it. Thus oncogenic Kras is required to generate CPS1 “addiction.” This addiction might result from the ability of mutant Kras to increase glutaminolysis in mitochondria (33) thus locally generating ammonia that would support carbamoyl phosphate production by CPS1 (9).

## Mutant KRAS in Lipid Metabolism

Lipid metabolism, in particular the synthesis of fatty acids, is required for membrane biosynthesis, signaling molecules production and energy storage (48). Recently, it is also emerging as a mechanism to cope with oncogenic stress (49). Mutant Kras has been shown to control both β-oxidation and *de novo* lipogenesis in NSCLC (49, 50). The role of mutant Kras in fatty acid oxidation has been reported in a transgenic mouse model that expresses the (doxy)-inducible Kras transgene (*Kras*^*G12D*^) in the respiratory epithelium (49). These mice, when fed with doxy, develop lung tumors that completely regress when doxycycline is removed with concomitant significant decrease in the expression of enzymes that control glycolysis and lipid metabolism (49). Among the latters, Acyl-coenzyme A synthetase long chain family member 3 and 4 (*Acsl3* and *Acsl4*) are significantly down regulated in tumors undergoing Kras^G12D^ extinction and *Acsl3* seems to contribute the most to the oncogenic phenotype both *in vitro* and *in vivo* (49). *Acsl3* promotes uptake, retention, and β-oxidation of fatty acids converting them into Acyl-CoA esters. Genetic deletion of *Acsl3* in mice does not cause any morphological defects neither during development nor adult life, but impairs mutant Kras tumorigenesis. *Acsl3* silencing has likely similar effects as fatty acid synthase pharmacological inhibition opening to new possible therapeutic strategies in NSCLS (49).

The role of Kras in lipogenesis is highlighted by the upregulation of enzymes that control fatty acid metabolism such as ATP citrate lyase, fatty acid synthase and acetyl coenzyme A carboxylase in the Kras^G12D^ lung cancer model (50). Overexpression of both ATP citrate lyase and fatty acid synthase correlates with poor survival and with increased lipogenesis as shown by the higher levels of newly synthetized palmitate and oleate (48, 50).

As for other metabolic adaptations, *KRAS* mutations work synergistically with additional genetic alterations in reprogramming lipid metabolism. In PDAC arising from intraductal papillary mucinous neoplasm (IPMNs), *KRAS* mutations are associated to a gain of function mutation on the gene *GNAS* (*GNAS*^*R201C*^) which encodes Gα_s_, the stimulatory subunit of heterotrimeric G proteins (10). *GNAS* mediates G-protein-coupled receptor (GPCR)-stimulated cAMP signaling, and its mutation has been identified in different tumor types (10). In double mutant mice carrying inducible *Gnas*^*R201C*^ expression and *Kras*^*G12D*^ mutation, *Gnas*^*R201C*^ promotes IPMN initiation and sustains tumor formation. Mechanistically, using tumor-derived organoids, *Gnas*^*R201C*^ has been found to support pancreatic cancer growth via cAMP-PKA signaling that suppresses the salt-inducible kinases (SIKs) (10). Proteomics reveals that this pathway is overall correlated with lipid metabolism and with components of the peroxisome, an organelle required for long-chain fatty acids processing and the generation of ether lipids suggesting that concurrent *GNAS* and *KRAS* mutations cooperate in lipid metabolism rewiring (10).

## Conclusions

Studies on the role of mutant Kras in rewiring cancer cell metabolism are blooming and the approaches to exploit Kras-driven metabolic vulnerabilities that stem from these findings hold promises, at least in pre-clinical settings, as we summarized in Table 1. A take home message is that metabolic interfering drugs can be attempted, preferentially in combination with other therapies, to tackle Kras mutant cancers but, to be successful, these strategies have to consider the genetic mutational background, the tissue of origin and the crosstalk between the tumor and the microenvironment. It is of note that some of the putative targets including AMPK and autophagy have, depending on the context, pro-tumorigenic functions, while others, such as ATF4, by regulating transcription of distinct set of genes, are endowed with a wide range of downstream functions. This could pose limits to their exploitation as therapeutic targets (23, 51, 52). Moreover, findings on the role of AMPK in Kras^G12D^-driven lung cancer during glucose starvation (25), and on the KRAS-dependent transcriptional response to nutrient deprivation (45), reveal that the effects of *KRAS* mutations on metabolic reprogramming are also strongly influenced by the availability of nutrients which can be heterogeneously distributed within the tumor and change over time. There is a lot more to be learned, there are still big research gaps in the field that need to be addressed in future studies. Moreover the interplay with other pathways, such as PPARγ and WNT/β-catenin, involved in metabolic enzymes changes in other cancers (53, 54) should be further investigated. This growing body of knowledge points to the complexity of this system and suggests that analysis of the genetic context and the metabolic activity of the tumor should be combined to identify KRAS-driven metabolic vulnerabilities and stratify patients.

**Table 1:** Summary of potential metabolic targets in PDAC and NSCLC.

**Cancer type**	**Potential metabolic targets**	**Proposed mechanism**	**Proposed inhibitor**	**References**
PDAC	Penthose phosphate pathway (PPP)	MAPK through Kras leads to an increase of glycolytic enzymes expression	PPP inhibition	(21)
PDAC	PON2	Suppresses cell detachment-induced cell death (anoikis) by inhibiting the AMPK/FOXO3A/PUMA pathway	Pharmacological inhibition of PON2 or activation of AMPK	(7, 23)
PDAC NSCLC	Tfeb/Tfe3	Tfe3 sustains tumor growth through increased lysosomal activity	Inhibition of lysosomal function	(25, 26)
PDAC	GOT1 and GOT2	Elevating the NADPH/NADP^+^ ratio leading to higher antioxidant capacity of tumor cells	GOT1 inhibition	(33, 34)
PDAC	MAPK (MEK1/2, ERK) and autophagy pathway	MAPK inhibition leads to tumor cell addiction to autophagy	Combined inhibition of autophagy and MAPK in cells addicted to autophagy	(43, 44)
NSCLC	ATF4 transcription factor	Amino acid dependency	Inhibition of glutamine utilization	(45)
NSCLC	Carbamoyl phosphate synthetase-1 (CPS1)	KRAS/LKB1 mutant enhances CPS1 expression, pyrimidine synthesis and glutaminolysis	Inhibition of CPS1 or glutamine utilization	(9, 33)
NSCLC	Acsl3	Kras enhances Acsl3 activity and lipid metabolism	Silencing or inhibition of Acsl3	(49)
PDAC	GNAS	Promotes cAMP/PKA signaling and metabolism rewiring	Inhibitors of the cAMP/PKA pathway and lipid metabolism	(10)

## Author Contributions

EP and LL wrote the manuscript. EP and EM performed the staining showed in Figure 1. DA designed Figure 2. FB and LL reviewed the final version of the manuscript.

### Conflict of Interest Statement

The authors declare that the research was conducted in the absence of any commercial or financial relationships that could be construed as a potential conflict of interest.

## Glossary

The use of capital letters or the italic to indicate KRAS reflects the

nomenclature guidelines here reported.

KRAS human protein.

*KRAS* human gene.

Kras murine protein.

*Kras* murine gene.

## References

[1] Kimmelman AC. Metabolic dependencies in RAS-driven cancers. Clin Cancer Res. (2015) 21:1828–34. 10.1158/1078-0432.CCR-14-2425 25878364 PMC4400826

[2] Yuneva MO Fan TW Allen TD Higashi RM Ferraris DV Tsukamoto T. The metabolic profile of tumors depends on both the responsible genetic lesion and tissue type. Cell Metab. (2012) 15:157–70. 10.1016/j.cmet.2011.12.015 22326218 PMC3282107

[3] Mayers JR Torrence ME Danai LV Papagiannakopoulos T Davidson SM Bauer MR. Tissue of origin dictates branched-chain amino acid metabolism in mutant Kras-driven cancers. Science. (2016) 353:1161–5. 10.1126/science.aaf5171 27609895 PMC5245791

[4] Tape CJ Ling S Dimitriadi M McMahon KM Worboys JD Leong HS Oncogenic KRAS regulates tumor cell signaling via stromal reciprocation. Cell. (2016) 165:910–20. 10.1016/j.cell.2016.03.029 27087446 PMC4868820

[5] Yang A Herter-Sprie G Zhang H Lin EY Biancur D Wang X. Autophagy sustains pancreatic cancer growth through both cell-autonomous and nonautonomous mechanisms. Cancer Discov. (2018) 8:276–87. 10.1158/2159-8290.CD-17-0952 29317452 PMC5835190

[6] Sousa CM Biancur DE Wang X Halbrook CJ Sherman MH Zhang L. Pancreatic stellate cells support tumour metabolism through autophagic alanine secretion. Nature. (2016) 536:479–83. 10.1038/nature19084 27509858 PMC5228623

[7] Nagarajan A Dogra SK Sun L Gandotra N Ho T Cai G. Paraoxonase 2 facilitates pancreatic cancer growth and metastasis by stimulating GLUT1-mediated glucose transport. Mol Cell. (2017) 67:685–701.e6. 10.1016/j.molcel.2017.07.014 28803777 PMC5567863

[8] Romero R Sayin VI Davidson SM Bauer MR Singh SX LeBoeuf SE. Keap1 loss promotes Kras-driven lung cancer and results in dependence on glutaminolysis. Nat Med. (2017) 23:1362–8. 10.1038/nm.4407 28967920 PMC5677540

[9] Kim J Hu Z Cai L Li K Choi E Faubert B. CPS1 maintains pyrimidine pools and DNA synthesis in KRAS/LKB1-mutant lung cancer cells. Nature. (2017) 546:168–72. 10.1038/nature22359 28538732 PMC5472349

[10] Patra KC Kato Y Mizukami Y Widholz S Boukhali M Revenco I. Mutant GNAS drives pancreatic tumourigenesis by inducing PKA-mediated SIK suppression and reprogramming lipid metabolism. Nat Cell Biol. (2018) 20:811–22. 10.1038/s41556-018-0122-3 29941929 PMC6044476

[11] Racker E Resnick RJ Feldman R Glycolysis and methylaminoisobutyrate uptake in rat-1 cells transfected with ras or myc oncogenes. Proc Natl Acad Sci USA. (1985) 82:3535–8. 10.1073/pnas.82.11.3535 3858838 PMC397819

[12] Hruban RH Wilentz RE Kern SE. Genetic progression in the pancreatic ducts. Am J Pathol. (2000) 156:1821–5. 10.1016/S0002-9440(10)65054-7 10854204 PMC1850064

[13] Guerra C Barbacid M. Genetically engineered mouse models of pancreatic adenocarcinoma. Mol Oncol. (2013) 7:232–47. 10.1016/j.molonc.2013.02.002 23506980 PMC5528418

[14] Xie H Hanai J Ren JG Kats L Burgess K Bhargava P. Targeting lactate dehydrogenase–a inhibits tumorigenesis and tumor progression in mouse models of lung cancer and impacts tumor-initiating cells. Cell Metab. (2014) 19:795–809. 10.1016/j.cmet.2014.03.003 24726384 PMC4096909

[15] Barron CC Bilan PJ Tsakiridis T Tsiani E. Facilitative glucose transporters: implications for cancer detection, prognosis and treatment. Metabolism. (2016) 65:124–39. 10.1016/j.metabol.2015.10.007 26773935

[16] Pinho AV Mawson A Gill A Arshi M Warmerdam M Giry-Laterriere M. Sirtuin 1 stimulates the proliferation and the expression of glycolysis genes in pancreatic neoplastic lesions. Oncotarget. (2016) 7:74768–78. 10.18632/oncotarget.11013 27494892 PMC5342700

[17] Basturk O Singh R Kaygusuz E Balci S Dursun N Culhaci N. GLUT-1 expression in pancreatic neoplasia: implications in pathogenesis, diagnosis, and prognosis. Pancreas. (2011) 40:187–92. 10.1097/MPA.0b013e318201c935 21206329 PMC3164314

[18] Guerra C Schuhmacher AJ Cañamero M Grippo PJ Verdaguer L Pérez-Gallego L. Chronic pancreatitis is essential for induction of pancreatic ductal adenocarcinoma by K-Ras oncogenes in adult mice. Cancer Cell. (2007) 11:291–302. 10.1016/j.ccr.2007.01.012 17349585

[19] Pupo E Ducano N Lupo B Vigna E Avanzato D Perera T. Rebound effects caused by withdrawal of MET kinase inhibitor are quenched by a MET therapeutic antibody. Cancer Res. (2016) 76:5019–29. 10.1158/0008-5472.CAN-15-3107 27364553

[20] DeBerardinis RJ Chandel NS. Fundamentals of cancer metabolism. Sci Adv. (2016) 2:e1600200. 10.1126/sciadv.1600200 27386546 PMC4928883

[21] Ying H Kimmelman AC Lyssiotis CA Hua S Chu GC Fletcher-Sananikone E. Oncogenic Kras maintains pancreatic tumors through regulation of anabolic glucose metabolism. Cell. (2012) 149:656–70. 10.1016/j.cell.2012.01.058 22541435 PMC3472002

[22] Santana-Codina N Roeth AA Zhang Y Yang A Mashadova O Asara JM. Oncogenic KRAS supports pancreatic cancer through regulation of nucleotide synthesis. Nat Commun. (2018) 9:4945. 10.1038/s41467-018-07472-8 30470748 PMC6251888

[23] Hardie DG Molecular pathways: is AMPK a friend or a foe in cancer? Clin Cancer Res. (2015) 21:3836–40. 10.1158/1078-0432.CCR-14-3300 26152739 PMC4558946

[24] Kimmelman AC White E. Autophagy and tumor metabolism. Cell Metab. (2017) 25:1037–43. 10.1016/j.cmet.2017.04.004 28467923 PMC5604466

[25] Eichner LJ Brun SN Herzig S Young NP Curtis SD Shackelford DB. Genetic analysis reveals AMPK is required to support tumor growth in murine Kras-dependent lung cancer models. Cell Metab. (2019) 29:285–302.e7. 10.1016/j.cmet.2018.10.005 30415923 PMC6365213

[26] Perera RM Stoykova S Nicolay BN Ross KN Fitamant J Boukhali M. Transcriptional control of autophagy-lysosome function drives pancreatic cancer metabolism. Nature. (2015) 524:361–5. 10.1038/nature14587 26168401 PMC5086585

[27] Sardiello M Palmieri M di Ronza A Medina DL Valenza M Gennarino VA. A gene network regulating lysosomal biogenesis and function. Science. (2009) 325:473–7. 10.1126/science.1174447 19556463

[28] Settembre C Di Malta C Polito VA Garcia Arencibia M Vetrini F Erdin S. TFEB links autophagy to lysosomal biogenesis. Science. (2011) 332:1429–33. 10.1126/science.1204592 21617040 PMC3638014

[29] Sigismund S Confalonieri S Ciliberto A Polo S Scita G Di Fiore PP. Endocytosis and signaling: cell logistics shape the eukaryotic cell plan. Physiol Rev. (2012) 92:273–366. 10.1152/physrev.00005.2011 22298658 PMC5614474

[30] Commisso C Davidson SM Soydaner-Azeloglu RG Parker SJ Kamphorst JJ Hackett S. Macropinocytosis of protein is an amino acid supply route in Ras-transformed cells. Nature. (2013) 497:633–7. 10.1038/nature12138 23665962 PMC3810415

[31] Recouvreux MV Commisso C. Macropinocytosis: a metabolic adaptation to nutrient stress in cancer. Front Endocrinol. (2017) 8:261. 10.3389/fendo.2017.00261 29085336 PMC5649207

[32] Gaglio D Soldati C Vanoni M Alberghina L Chiaradonna F. Glutamine deprivation induces abortive s-phase rescued by deoxyribonucleotides in k-ras transformed fibroblasts. PLoS ONE. (2009) 4:e4715. 10.1371/journal.pone.0004715 19262748 PMC2650790

[33] Son J Lyssiotis CA Ying H Wang X Hua S Ligorio M. Glutamine supports pancreatic cancer growth through a KRAS-regulated metabolic pathway. Nature. (2013) 496:101–5. 10.1038/nature12040 23535601 PMC3656466

[34] Duan G Shi M Xie L Xu M Wang Y Yan H. Increased glutamine consumption in cisplatin-resistant cells has a negative impact on cell growth. Sci Rep. (2018) 8:4067. 10.1038/s41598-018-21831-x 29511244 PMC5840399

[35] Kerr EM Gaude E Turrell FK Frezza C Martins CP. Mutant Kras copy number defines metabolic reprogramming and therapeutic susceptibilities. Nature. (2016) 531:110–3. 10.1038/nature16967 26909577 PMC4780242

[36] Pavlova NN Thompson CB. The emerging hallmarks of cancer metabolism. Cell Metab. (2016) 23:27–47. 10.1016/j.cmet.2015.12.006 26771115 PMC4715268

[37] Lock R Roy S Kenific CM Su JS Salas E Ronen SM. Autophagy facilitates glycolysis during Ras-mediated oncogenic transformation. Mol Biol Cell. (2011) 22:165–78. 10.1091/mbc.e10-06-0500 21119005 PMC3020913

[38] Guo JY Chen HY Mathew R Fan J Strohecker AM Karsli-Uzunbas G. Activated Ras requires autophagy to maintain oxidative metabolism and tumorigenesis. Genes Dev. (2011) 25:460–70. 10.1101/gad.2016311 21317241 PMC3049287

[39] Guo JY Teng X Laddha SV Ma S Van Nostrand SC Yang Y. Autophagy provides metabolic substrates to maintain energy charge and nucleotide pools in Ras-driven lung cancer cells. Genes Dev. (2016) 30:1704–17. 10.1101/gad.283416.116 27516533 PMC5002976

[40] Lock R Kenific CM Leidal AM Salas E Debnath J. Autophagy-dependent production of secreted factors facilitates oncogenic RAS-driven invasion. Cancer Discov. (2014) 4:466–79. 10.1158/2159-8290.CD-13-0841 24513958 PMC3980002

[41] Amaravadi R Debnath J. Mouse models address key concerns regarding autophagy inhibition in cancer therapy. Cancer Discov. (2014) 4:873–5. 10.1158/2159-8290.CD-14-0618 25092744 PMC4124512

[42] Yang A Rajeshkumar NV Wang X Yabuuchi S Alexander BM Chu GC. Autophagy is critical for pancreatic tumor growth and progression in tumors with p53 alterations. Cancer Discov. (2014) 4:905–13. 10.1158/2159-8290.CD-14-0362 24875860 PMC4125497

[43] Kinsey CG Camolotto SA Boespflug AM Guillen KP Foth M Truong A Protective autophagy elicited by RAF → MEK → ERK inhibition suggests a treatment strategy for RAS-driven cancers. Nat Med. (2019) 25:620–7. 10.1038/s41591-019-0367-9 30833748 PMC6452642

[44] Bryant KL Stalnecker CA Zeitouni D Klomp JE Peng S Tikunov AP. Combination of ERK and autophagy inhibition as a treatment approach for pancreatic cancer. Nat Med. (2019) 25:628–40. 10.1038/s41591-019-0368-8 30833752 PMC6484853

[45] Gwinn DM Lee AG Briones-Martin-Del-Campo M Conn CS Simpson DR Scott AI. Oncogenic KRAS regulates amino acid homeostasis and asparagine biosynthesis via ATF4 and alters sensitivity to L-asparaginase. Cancer Cell. (2018) 33:91–107.e6. 10.1016/j.ccell.2017.12.003 29316436 PMC5761662

[46] Sullivan LB Gui DY Vander Heiden MG. Altered metabolite levels in cancer: implications for tumour biology and cancer therapy. Nat Rev Cancer. (2016) 16:680–93. 10.1038/nrc.2016.85 27658530

[47] Morris SM. Regulation of enzymes of the urea cycle and arginine metabolism. Annu Rev Nutr. (2002) 22:87–105. 10.1146/annurev.nutr.22.110801.140547 12055339

[48] Röhrig F Schulze A. The multifaceted roles of fatty acid synthesis in cancer. Nat Rev Cancer. (2016) 16:732–49. 10.1038/nrc.2016.89 27658529

[49] Padanad MS Konstantinidou G Venkateswaran N Melegari M Rindhe S Mitsche M. Fatty acid oxidation mediated by Acyl-CoA synthetase long chain 3 is required for mutant KRAS lung tumorigenesis. Cell Rep. (2016) 16:1614–28. 10.1016/j.celrep.2016.07.009 27477280 PMC4981512

[50] Singh A Ruiz C Bhalla K Haley JA Li QK Acquaah-Mensah G *De novo* lipogenesis represents a therapeutic target in mutant Kras non-small cell lung cancer. FASEB J. (2018) 32:7018–27. 10.1096/fj.201800204 PMC6219836 29906244

[51] Nazio F Bordi M Cianfanelli V Locatelli F Cecconi F. Autophagy and cancer stem cells: molecular mechanisms and therapeutic applications. Cell Death Differ. (2019) 26:690–702. 10.1038/s41418-019-0292-y 30728463 PMC6460398

[52] Morselli E Galluzzi L Kepp O Vicencio JM Criollo A Maiuri MC. Anti- and pro-tumor functions of autophagy. Biochim Biophys Acta. (2009) 1793:1524–32. 10.1016/j.bbamcr.2009.01.006 19371598

[53] Lecarpentier Y Claes V Vallée A Hébert JL. Thermodynamics in cancers: opposing interactions between PPAR gamma and the canonical WNT/beta-catenin pathway. Clin Transl Med. (2017) 6:14. 10.1186/s40169-017-0144-7 28405929 PMC5389954

[54] Lemieux E Cagnol S Beaudry K Carrier J Rivard N. Oncogenic KRAS signalling promotes the Wnt/β-catenin pathway through LRP6 in colorectal cancer. Oncogene. (2015) 34:4914–27. 10.1038/onc.2014.416 25500543 PMC4687460

